# Safety and Efficacy of Zero-Profile Polyetheretherketone (PEEK) Cages Filled with Biphasic Calcium Phosphate (BCP) in Anterior Cervical Discectomy and Fusion (ACDF): A Case Series

**DOI:** 10.3390/jcm13071919

**Published:** 2024-03-26

**Authors:** Marco Battistelli, Edoardo Mazzucchi, Mario Muselli, Gianluca Galieri, Filippo Maria Polli, Fabrizio Pignotti, Alessandro Olivi, Giovanni Sabatino, Giuseppe La Rocca

**Affiliations:** 1 Department of Neurosurgery, Fondazione Policlinico Universitario A. Gemelli IRCCS, 00168 Rome, Italy; marco.battistelli23494@gmail.com (M.B.); filippomaria.polli@policlinicogemelli.it (F.M.P.); alessandro.olivi@policlinicogemelli.it (A.O.);; 2 Department of Neurosurgery, Mater Olbia Hospital, 07026 Olbia, Italy; edoardo.mazzucchi@gmail.com (E.M.);; 3 Department of Life, Health and Environmental Sciences, University of L’Aquila, 67100 L’Aquila, Italy; mariomuselli@uniaq.it

**Keywords:** case series, ACDF, zero-profile PEEK, biphasic calcium phosphate (BCP), subsidence, PROMs

## Abstract

**Background**: In the evolving landscape of anterior cervical discectomy and fusion (ACDF), the integration of biomechanical advancements and proper fusion-enhancing materials is crucial for optimizing patient outcomes. This case series evaluates the efficacy and clinical implications of employing zero-profile polyetheretherketone (PEEK) cages filled with biphasic calcium phosphate (BCP) in ACDF procedures, focusing on fusion and subsidence rates alongside patient disability, residual pain, and quality of life. **Methods**: This case series comprises 76 consecutive patients, with a median follow-up of 581 days. The Bridwell classification system was used for assessing fusion rates while subsidence occurrence was recorded, correlating these radiographic outcomes with clinical implications. **Results**: The results demonstrated a satisfactory fusion rate (76.4% for grades I and II). The subsidence rate was low (6.74% of segments). Significant clinical improvements were observed in pain, disability, and quality-of-life metrics, aligning with the minimum clinically important difference thresholds; however, subgroup analyses demonstrated that subsidence or pseudoarthrosis group improvement of PROMs was not statistically significant with respect to baseline. ANOVA analyses documented that subsidence has a significant weight over final follow-up pain and disability outcomes. No dysphagia cases were reported. **Conclusions**: These findings underscore the efficacy of zero-profile PEEK cages filled with BCP in ACDF, highlighting their potential to improve patient outcomes while minimizing complications. Pseudoarthrosis and subsidence have major implications over long-term PROMs. The study reinforces the importance of selecting appropriate surgical materials to enhance the success of ACDF procedures.

## 1. Introduction

Anterior cervical discectomy and fusion (ACDF) was introduced in the 1950s as a surgical solution for cervical disc degeneration [1,2]. It was Robinson and Smith who first described anterior cervical fusion. Over the last 80 years, pioneering surgeons have continuously advanced ACDF techniques through the introduction of increasingly sophisticated surgical instrumentation and devices. Today, anterior cervical decompression and fusion techniques have become the primary approaches for addressing cervical radiculopathy and myelopathy, eliminating the need for orthosis to maintain spine stability after the operation [3]. These advancements are grounded in a deeper understanding of cervical biomechanics, the origins of complications, and the characteristics of materials. With the increasing costs of health-related care, primarily due to the length of hospital stay (LOS), spine surgeons are seeking technological solutions to maintain satisfying outcomes while reducing the rate of complications and the need for reoperation [4,5].

Autologous bone graft harvested from the iliac crest has been the graft material of choice used in spinal fusion surgery because of its osteogenic, osteoinductive, and osteoconductive properties, in addition to being histocompatible and completely osteointegrative. However, it remains a technique with significant morbidity, mostly due to donor site morbidity. With the advent of structural allografts, the continued practice of using autologous bone grafts warrants further scrutiny [6]. Among cage materials gaining popularity, polyetheretherketone (PEEK) is gaining increasing interest. In vivo animal studies have investigated its biomechanical properties and identified a bone-like elastic modulus and radiolucency, which offers optimal bone integration and reduced radiographic artifacts [7]. However, because allografts lack significant osteoinductive and osteoconductive properties, they can be insufficient for achieving optimal fusion. Thus, allografts are commonly augmented with synthetic bone graft substitutes. Among them, biphasic calcium phosphate (BCP) has shown peculiar bone remodeling properties. A histological study by Yamada et al. aimed at assessing which bone graft substitute was the most conducive to osteoclastic activity, documenting BCP’s ability to inhibit osteoclasts thanks to calcium and the formation of resorption lacunae similar to those on normal bone. This suggests BCP to be a more natural surface than other bone graft substitutes [8].

Another innovation in cervical spine cages is represented by the introduction of zero-profile devices. Zero-profile cages show lower subsidence and the better restoration of cervical lordosis and disc height compared to standalone devices [9]. Since a decreased C2-C7 Cobb angle and postoperative kyphotic deformity have been correlated with a higher incidence of clinical adjacent segment disease (ASD) requiring reoperation and higher disability, they have the potential to offer significant advantages over standalone ones [10].

In this context, some authors have investigated the role of zero-profile PEEK cages filled with BCP [11,12,13,14]. They have reported a shorter hospital stay and operative time, reduced blood loss, and optimal fusion with this peculiar cage material, design, and bone substitute. However, a gap has emerged regarding the evaluation of subsidence incidence and pseudoarthrosis and subsidence clinical impact on patients. The present study aims to investigate the safety and clinical results of ACDF performed using zero-profile cages filled with BCP. An emphasis is placed on the frequency of subsidence and pseudoarthrosis occurrence and their clinical implications on patients’ disability and quality of life.

## 2. Materials and Methods

We conducted a retrospective review of prospectively collected data on patients who underwent ACDF with zero-profile interlock PEEK cages (CoRoent^®^ Small InterlockTM system, NuVasive^®^, San Diego, CA, USA) between July 2019 and November 2021 at our institution. Indications for surgery included cervical spondylosis confirmed by MRI, whose neurologic symptoms were unresponsive to at least six weeks of conservative therapies. Patients underwent preoperative MRI, a one-day post-operative X-ray, and a follow-up (FU) X-ray executed at least one year after the surgery. Exclusion criteria were less than one year FU, prior cervical procedures, use of devices other than zero-P interlock PEEK cages with 7° lordosis packed with a biphasic calcium–phosphate bone graft substitute, or unwillingness to undergo follow-up questionnaires and/or X-ray.

### 2.1. Clinical and Radiological Data

We collected data preoperatively, one month post operatively, and at least one year post operatively, including the Visual Analogue Scale (VAS) [15], Short Form-36 Score Health Survey (SF-36) [16], Neck Disability Index (NDI) [17], Oswestry Disability Index (ODI) [18], EuroQol-5D (EQ-5D) [19], subsidence rate, and fusion rate according to the Bridwell classification system [20]. The aesthetic was evaluated with a subjective scale, where 1 was very satisfied, 2 was satisfied, 3 was moderately satisfied, and 4 was unsatisfied with wound aesthetic. The fusion was ascertained via a plain X-ray [21]. Subsidence was defined as a >2 mm reduction in disc height or cage migration [22]. Two independent surgeons assessed radiographic fusion and subsidence, with discrepancies resolved by a third senior operator. Demographic and risk factors, such as age, gender, body mass index (BMI), hypertension, diabetes mellitus (DM), fibromyalgia, and smoking, were assessed.

### 2.2. Surgical Procedure and Cage

**Figure 1:**
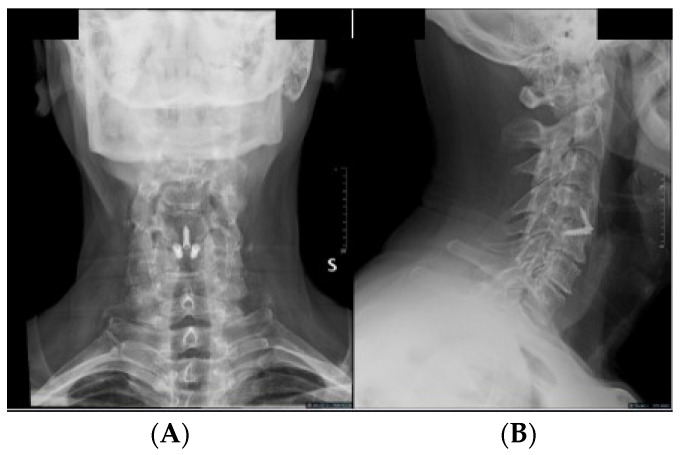
Postoperative (**A**) AP and (**B**) lateral X-ray displaying a C4-5 ACDF using an interfixated zero-profile PEEK cage.

All procedures were performed by a senior experienced surgeon (G.S.). A standard anterior Cloward approach was employed [1], with Caspar distractors used as needed for vertebral body distraction. The discectomy and vertebral plate preparation were performed using high-speed drills and/or curettes, ensuring posterior longitudinal ligament opening and neural decompression. We used zero-P interlock PEEK cages with 7° lordosis, packed with a biphasic calcium–phosphate bone graft substitute (AttraXPutty^®^, NuVasive^®^, San Diego, CA, USA). Three screws anchored the cage, with two in the superior vertebral body and one in the inferior one at a 40° angle (Figure 1).

### 2.3. Ethical Approval

All patients provided written informed consent to the surgical procedure and to the collection of data, and the study received approval from the local ethics committee.

### 2.4. Statistical Analysis

Data analysis utilized STATA-15-BE. Spearman’s correlation examined associations between ODI, NDI, VAS, EQ-5D, SF-36(PS), and SF-36(MS). Changes in these parameters were assessed using the Friedman test. Two-way ANOVA evaluated associations between HRQoL outcomes and fusion and subsidence status. The significance was defined as *p* < 0.05.

## 3. Results

### 3.1. Demographic Data (Table 1)

Between July 2019 and November 2021, one hundred and eleven consecutive patients underwent ACDF at our institution. Three patients were excluded: (1) two of them were excluded because tantalum cages were used, and (2) one had C7-Th1 ACDF. Thirty-two patients declined to undergo follow-up X-rays and/or complete follow-up questionnaires, and consequently were excluded from the analysis. Seventy-six patients were finally included in the study, with a mean follow-up (FU) of 638.3 ± 216.7 days. The mean age was 51.8 ± 10.0 years, with thirty-two men (42.1%) and forty-four women (57.9%). Twelve patients had multiple level ACDF, and the mean BMI was 25.5 ± 4.4 kg/m^2^. Hypertension was present in 19 (25.0%) patients, 7 (9.2%) had diabetes mellitus (DM), 27 (35.5%) were active smokers, and 4 (5.3%) had fibromyalgia.

**Table 1:** Demographic and clinical data.

Age (years), median (IQR)	51.5 (13.5)
Gender, n (%)	
Male	32 (42.1%)
Female	44 (57.9%)
BMI (kg/m^2^), median (IQR)	26.4 (3.9)
Hypertension (%)	19 (25.0%)
Diabetes mellitus (%)	7 (9.2%)
Fibromyalgia (%)	4 (5.3%)
Smoke (%)	27 (35.5%)
Follow-up (days), median (IQR)	581 (413)
Single/multilevel surgery	64/12

### 3.2. Pain, Function Disability, and Quality of Life (Table 2)

Preoperatively, the average VAS was 7.3 ± 1.8, which significantly decreased to 3.1 ± 2.3 at one month and 3.6 ± 2.8 at the final FU (*p* < 0.001). SF-36(PS) and SF-36(MS) also improved significantly between preoperative and one-month and final FU assessments. Mean SF-36(MS) was 54.8 ± 19.9 preoperatively, 63.5 ± 17.1 one month postoperatively, and 62.2 ± 20.9 at the final FU (*p* = 0.002). Similarly, mean SF-36(PS) was 45.2 ± 19.2 preoperatively, 57.8 ± 20.7 one month postoperatively, and 58.3 ± 23.9 at the final FU (*p* = 0.002). Quality-of-life parameters, including EQ-5D, showed significant improvements at the final FU; it was 0.5 ± 0.2 preoperatively and 0.7 ± 0.2 at both one month and the final FU (*p* < 0.001). The ODI decreased from 0.4 ± 0.2 (%) preoperatively to 0.2 ± 0.2 (%) at both one month and the final FU (*p* < 0.0001). The NDI improved from 0.6 ± 0.3 preoperatively to 0.3 ± 0.3 at both one month and FU (*p* < 0.0001). No patients reported dysphagia at one month and the final FU evaluations. The wound aesthetic mean value was 1.6 ± 0.8 at the one-month measurement.

**Table 2:** Outcome measures of pain, disability, quality-of-life parameters, and aesthetic and fusion parameters.

Variable	Mean	SD	*p*-Value
Preop-VAS *	7.3	±1.8	<0.0001
1-M-VAS	3.1	±2.3	
FU-VAS	3.6	±2.8	
Preop-EQ-5D *	0.50	±0.20	<0.0001
1-M-EQ-5D	0.70	±0.20	
FU-EQ-5D	0.70	±0.20	
Preop-SF-36(MS) *	54.8	±19.9	0.002
1-M-SF-36(MS)	63.5	±17.1	
FU-SF-36(MS)	62.2	±20.9	
Preop-SF-36(PS) *	45.2	±19.2	0.002
1-M-SF-36(PS)	57.8	±20.7	
FU-SF-36(PS)	58.3	±23.9	
Preop-ODI *	0.40	±0.20	<0.0001
1-M-ODI	0.20	±0.20	
FU-ODI	0.20	±0.20	
Preop-NDI *	0.60	±0.20	<0.0001
1-M-NDI	0.30	±0.20	
FU-NDI	0.30	±0.20	
Aesthetic ^1^	1.6	±0.8	
Fusion ^2^	1.8	±1.1	
1-M Dysphagia	0	-	
FU Dysphagia	0	-	

Values are presented as mean ± standard deviation. Statistical significance was reached if the *p*-value between the values was less than 0.05. Difference between preoperative and one-month post-operative values was statistically significant for all pain, disability, and quality-of-life parameters. ^1^ Aesthetic is reported as mean ± standard deviation of the values obtained from a questionnaire in which patients expressed their satisfaction over the aesthetic of the surgical cut as follows: (1) excellent, (2) good, (3) moderately satisfactory, (4) poor. ^2^ Fusion is reported as mean ± standard deviation of the values obtained from evaluation of final follow-up X-ray according to Bridwell classification. * VAS: Visual Analogue Scale; SF-36(PS): Short Form-36 Score Health Survey Physical Score; SF-36(MS): Short Form-36 Score Health Survey Mental Score; NDI: Neck Disability Index (NDI); ODI: Oswestry Disability Index; EQ-5D: EuroQol-5D.

### 3.3. Radiographic Outcome (Table 2)

The fusion rate was as follows: 55.3% grade I, 21.1% grade II, 9.2% grade III, and 10.5% grade IV (mean 1.8 ± 1.1). In multilevel ACDF, 75% achieved grade I, while 8.33% and 16.67% scored grade II and III, respectively. In the single-level group, 51.5% scored grade I, while 23.4%, 7.8%, and 15.6% scored grade II, III, and IV, respectively. Subsidence occurred in 6.74% of the segments, none of which were in the multilevel ACDF group. The distribution of subsidence was as follows: three cases on C4C5, three cases on C5C6, and one case on C6C7. Only one patient required revision surgery due to symptomatic adjacent segmental disease (ASD). At the final FU X-ray, two patients reported a screw fracture without related symptoms.

### 3.4. Correlation between Radiological and Clinical Outcomes (Table 3 and Table 4)

Mean values of pain, disability, and quality-of-life parameters preoperatively and at the final follow-up highlighted that all parameters improved in the non-subsidence and fusion group (*p* < 0.05). On the contrary, only the VAS improved statistically significantly at the last follow-up in the non-fusion group (*p* = 0.0138). In the subsidence group, no parameters showed a significant improvement (Table 3).

In view of these results, we decided to evaluate the “weight” of subsidence and fusion status on final pain, disability, and quality-of-life parameters through ANOVA tests. ANOVA analyses demonstrated statistically significant correlations between ODI and VAS with the subsidence status (ANOVA *p* = 0.044 and *p* = 0.005, respectively). No statistically significant correlations were observed with NDI, EQ-5D, and SF-36(PS). The fusion status did not significantly correlate with ODI, VAS, NDI, EQ-5D, and SF-36(PS). However, there was a trend towards significance for the VAS in relation to the fusion status (ANOVA *p* = 0.092).

**Table 3:** Mean pain, disability, and quality of life preoperatively and at final FU stratified with respect of fusion and subsidence status.

	Non-Subsidence	Subsidence	Non-Fusion	Fusion
Preoperative ODI	0.40 (0.20)	0.30 (0.20)	0.40 (0.20)	0.40 (0.20)
FU ODI	0.20 (0.20)	0.40 (0.30)	0.30 (0.30)	0.20 (0.20)
*p*-value	0.0001	0.676	0.2087	0.0001
Preoperative VAS	7.50 (1.70)	6.00 (2.30)	6.60 (2.30)	7.50 (1.60)
FU VAS	3.40 (2.70)	6.20 (3.00)	4.60 (3.40)	3.40 (2.60)
*p*-value	0.0001	0.3524	0.0138	0.0001
Preoperative NDI	0.60 (0.20)	0.60 (0.20)	0.60 (0.20)	0.60 (0.20)
FU NDI	0.30 (0.20)	0.50 (0.30)	0.40 (0.30)	0.30 (0.20)
*p*-value	0.0001	0.6895	0.1135	0.0001
Preoperative ED_5D	0.50 (0.20)	0.60 (0.20)	0.50 (0.20)	0.50 (0.20)
FU ED_5D	0.70 (0.20)	0.50 (0.20)	0.60 (0.300)	0.70 (0.20)
*p*-value	0.0001	0.987	0.2219	0.0001
Preoperative SF-36 (PS)	44.7 (18.9)	52 (24.8)	45.8 (19.7)	45.1 (19.3)
FU SF-36 (PS)	59.9 (23.5)	42.9 (25.9)	49.8 (30.2)	60.9 (21.8)
*p*-value	0.0001	0.7837	0.5149	0.0002

Values are presented as mean (standard deviation). Fusion was considered as Bridwell grades I and II, while no-fusion was considered as Bridwell grade III and IV. In the non-subsidence and fusion groups, we observed a significant reduction in ODI, VAS, and NDI scores; differently, ED-5D and SF-36 (PS) scores increased significantly. In the subsidence group, we did not observe significant changes in any of the scales. In the non-fusion group, only the VAS decreased significantly.

**Table 4:** Pain, disability, and quality-of-life outcomes in dependence of fusion and subsidence status.

Group	Factor	Test of Between-Subjects Effects ^1^	Test of Within-Subjects Effects—Factor ^2^	Test of Within-Subjects Effects—Interaction ^3^
Subsidence	ODI	0.347	0.026	0.044
	VAS	0.368	<0.0001	0.005
	NDI	0.077	<0.0001	0.134
	EQ-5D	0.125	0.134	0.270
	SF-36(PS)	0.810	<0.0001	0.974
Fusion	ODI	0.359	<0.0001	0.227
	VAS	0.685	<0.0001	0.092
	NDI	0.229	<0.0001	0.865
	EQ-5D	0.304	<0.0001	0.689
	SF-36(PS)	0.908	<0.0001	0.975

ANOVA analyses. ^1^ Test of between-subjects effects expresses variations in the subject (i.e., ODI) with respect to the group (i.e., subsidence yes vs. subsidence no); if the *p*-value is <0.05, it can be inferred that there is a difference dependent upon the group (i.e., subsidence yes vs. subsidence no). ^2^ Test of between-subjects effects—factor shows the variation source that can be attributed to the interaction between factors (every measurement) into a certain subject (i.e., ODI); if *p*-value is <0.05, it can be inferred that there is a statistically significant difference between the measurements independently of the group (i.e., subsidence yes vs. subsidence no). ^3^ Test of within-subjects effects—interaction shows the variation source that can be attributed to the interaction between factor and group. If *p*-value is <0.05, it can be inferred that the difference between the repetitive measurements depends on the belonging of that factor to a certain group (i.e., subsidence yes vs. subsidence no).

## 4. Discussion

Surgical practice in ACDF has significantly evolved from the introduction of this technique thanks to the understanding of biomechanical implications on patients’ outcome. This has led biomechanical engineers to develop sophisticated cages and bone graft substitutes with the aim of guaranteeing the devices’ integration with bone surface, reducing the incidence of subsidence and the rate of pseudoarthrosis. Another important aspect of cervical devices to be considered is the incidence of complications and discomfort. In this context, as shown by a comparative case series from Chen et al., ACDF with plating have shown optimal results in terms of non-union, the loss of cervical lordosis, the loss in the fused angle, the loss of disc height, and subsidence incidence when compared to standalone PEEK cages with a follow-up of 24 months [23]. However, conventional cage and plate constructs show a worrisome incidence of dysphagia and adjacent segment disease (ASD) which have led to the introduction and increasing employment of zero-profile cages [24]. A systematic review and meta-analyses from Kahaer et al. confronted the clinical and radiographic outcomes of single-level ACDF performed with zero-profile cages with respect to cage–plate devices on 1866 patients. It emerged that both devices were effective in restoring radiographic parameters of the cervical spine in terms of the segmental Cobb angle, the cervical Cobb angle, subsidence, implant failure, and fusion. Similarly, clinical outcomes were comparable when evaluating NDI, VAS, SF-36, and mJOA. However, the patients who underwent ACDF using zero-profile cages had a significantly reduced operative time and intraoperative blood loss, as well as the risk of ASD and of short-term, medium-term, and long-term dysphagia. In this view, the authors recommended zero-profile cages when a single-level ACDF is to be performed [25]. Similarly, zero-profile cages exhibit distinctive radiographic advantages with respect to standalone devices [9], which have led to their increasing popularity.

In the field of materials aimed at increasing fusion, many adjuvants have been investigated. BCP is a synthetic bone graft substitute made of a mixture of hydroxyapatite and β-tricalcium phosphate in fixed ratios. Yamada et al. conducted a histologic study on neonatal rabbit bone cells cultured for 2 days on hydroxyapatite and β-tricalcium phosphate (β-TCP) and two types of biphasic calcium phosphate (BCP) with HA/P-TCP ratios of 25/75 and 75/25. The authors were able to demonstrate osteoclasts’ activity on HA/P-TCP ratios of 25/75 culture which resembled that on human bone. This led the authors to affirm that BCP can co-exist with living bone tissue because it receives the natural action of bone cells during the remodeling process. However, because HA/P-TCP ratios of 25/75 present high solubility and low mechanical strength, to serve as an appropriate bone substitute, BCP 60/40 is normally used for clinical application due to its compromise between solubility and mechanical strength [26]. Cho et al. randomly divided 100 patients undergoing ACDF in two groups of 50 patients, of whom one received PEEK cages filled with BCP and the other PEEK cages filled with autologous bone. The fusion rates were 100% in both groups at 6 months as assessed with standard, flexion–extension, and bilateral oblique X-ray [12]. Thus, it can be affirmed that BCP can achieve fusion as effectively as autologous bone and that a 6-month follow-up is sufficient to evaluate this radiographic outcome. Despite these theoretical advantages, a few studies evaluated clinical applications of ACDF performed with zero-profile cages filled with BCP [11,12,13,14]. The outcome analyzed in those studies evaluated clinical results, in terms of disability, pain and quality-of-life parameters, and radiographic outcomes, in terms of fusion rate, while subsidence was not evaluated.

In light of these findings, we conducted a case series on 76 consecutive patients who underwent ACDF with zero-profile PEEK cages filled with BCP, aiming at assessing fusion and subsidence rates and their clinical implication on patients’ disability, residual pain, and quality of life. The peculiarity of this study is the evaluation of subsidence rate with this specific device, which was not evaluated in previous studies, and the statistical analyses of correlation between radiographic and clinical findings to evaluate the clinical consequences of pseudoarthrosis and/or subsidence with a long follow-up (median FU: 581 days; IQR: 413 days). At the final FU, a satisfactory fusion rate was achieved, which was evaluated as the absence of lucency around the cage (Bridwell classification system: 76.4% for grade I and II). Interestingly, the fusion rate was higher when the multilevel ACDF fusion rate was evaluated (Bridwell classification system: 83.33% grade I and II). The fusion rate in our case series appears inferior with respect to the previous ones. A retrospective cohort study from Maharaj et al. [11] conducted upon zero-profile PEEK cages filled with BCP highlighted that 21 out of 22 patients had a solid fusion at 9 months FU. In our case series, we employed a strict evaluation of fusion using criteria established by the Bridwell classification system, while Maharaj et al. employed a subjective method based on the extent of bridging bone, loss of radiolucency, restoration of interbody space, and absence of hardware failure, as evaluated by a radiologist. Thus, the lower fusion rate in our case series may be explained by variable fusion evaluation across the literature.

Radiographic outcomes in our report were satisfactory also in terms of overall subsidence incidence (subsidence rate: 6.74% of segments), while no cases were reported when considering the multilevel group. No previous studies are reported which evaluate subsidence incidence using zero-profile PEEK cages filled with BCP. The results regarding fusion and subsidence rate in the multilevel ACDF group raise concerns of a selection bias in the multilevel ACDF group, related to factors such as smoking status [27] and osteoporosis [28]. Stratifying patients based on the influence of these factors on radiographic outcome of ACDFs performed with the specific device we employed was not the object of this case series, but further research is encouraged to dig into these aspects.

When evaluating clinical results, in line with previous results reported throughout the literature [11,12,13,14], improvements in pain, disability, and quality-of-life parameters were significant with respect to the baseline. The reported improvement of pain, disability, and quality-of-life parameters satisfies the thresholds established by the minimum clinically important difference (MCID) according to the North American Spine Society (NASS) patient satisfaction scale for ACDF (2.6 points for VAS-NP, 4.1 points for VAS-AP, 17.3% for NDI, 8.1 points for SF-12(PS), 4.7 points for SF-12(MS), and 0.24 QALY for EQ-5D) [29]. Thus, ACDF performed with zero-profile cages filled with BCP proves as an effective treatment in improving patients’ pain, disability, and quality of life. However, when patients were stratified based on fusion and subsidence status, we observed significant clinical improvement in fusion and non-subsidence groups, while those who presented subsidence and pseudoarthrosis did not have a significant clinical improvement at the final follow-up, with the exception of the VAS in the subsidence group (Table 3). This represents a major result of the present case series since many authors agree on the absence of relevance of subsidence on clinical outcomes [30]. Moreover, these results show the relevance of subsidence and fusion on the success of surgery. In order to underscore the importance of these radiographic parameters on clinical outcomes, we conducted an ANOVA test to weigh the impact of fusion and subsidence on long-term surgery clinical success. We demonstrated a significant impact of subsidence on final FU VAS (*p* = 0.005) and ODI (*p* = 0.044), while fusion showed only a tendency towards impact on pain (*p* = 0.092). These results highlight the relevance of addressing the best surgical practices in order to avoid subsidence. Even if the measurement of cervical and segmental lordosis was above the scope of this case series, this finding can be explained by the fact that subsidence after ACDF produce a loss of segmental lordosis [31]. Since a lower C2-C7 Cobb angle and kyphotic deformity are related with higher disability [10], the subgroup of patients which suffered from subsidence in our study may have reached a degree of loss of segmental lordosis which produced a worse pain and disability outcome. However, the rate of subsidence using zero-profile cages filled with BCP is low enough that a high champion numerosity is necessary to avoid biases in the statistical analyses related to specific characteristics of the patients comprised in the subsidence subgroup. As for the fusion impact on clinical outcome, a recent retrospective cohort study from Lee et al. highlighted that the non-union group exhibited worse outcomes in terms of neck pain VAS, arm pain VAS, and NDI with respect to the union group at 5 years [32]. Thus, our results appear in line with recent evidence from the literature. Our case series is limited by a shorter follow-up and by included population numerosity. Since there is a clear tendency that fusion status have a major impact on the final follow-up pain, a more numerous population may have led to the detection of significancy.

In the field of patients’ comfort, our results demonstrated the absence of reported dysphagia in the short and long term and patients’ satisfaction with wound aesthetic. Dysphagia incidence is to be related to the cage design, and it has wildly been demonstrated that its incidence is more frequent with cage–plate constructs than zero-profile ones [24].

The strengths of this case report stand on the numerosity of the population included, which appears to be higher with respect to previous reports on this specific cage type [11,12,13,14], its monocentric nature, and the fact that all the surgical procedures were performed by the same senior author (G.S.) and can be considered homogeneous.

### Limitations

A relevant element in the choice of the implant to be used is cost-effectiveness, and it was not evaluated in the present study. The monocentric nature of the study could be considered a limitation of the generalizability of the results, but it is also, in our opinion, a strength of the present study, limiting the source of bias due to several surgeons using variable surgical techniques in different contexts of care. A longer FU in a larger population could be able to detect a higher rate of adjacent segment disease, which could be underreported in our cohort of patients. This will be the object of future studies. Smoking’s impact on fusion, subsidence, and HRQoL has not been evaluated as it would contribute minimally to the existing literature [33,34].

## 5. Conclusions

The use of zero-profile PEEEK cages filled with BCP in ACDF procedures has demonstrated promising radiographic and clinical results in terms of fusion, subsidence, and improvement in patients’ pain, disability, and quality of life, while dysphagia incidence is unremarkable. Although the fusion rate was slightly lower compared to previous studies, the significant clinical improvements observed confirm the effectiveness of this technique. Subgroup analyses demonstrated that a lack of fusion or the presence of subsidence negatively affects long-term PROMs. The study also revealed a notable implication between subsidence and worse pain and disability outcomes, underscoring the importance of achieving and maintaining proper segmental alignment and stability. These findings suggest that zero-profile PEEK cages with BCP are a viable option for ACDF, offering a balance between biomechanical stability and reduced operative morbidity. Further research is encouraged to explore the long-term effects of subsidence and the potential benefits of this approach in multilevel ACDF surgeries.

## Data Availability

The senior author possesses all the data and iconographic material that will be provided if requested.
